# Identification of circCIAO1(5) and circMALAT1 as Novel Potential Biomarkers for Bladder Cancer Monitoring Based on the Binding to miR-101-3p

**DOI:** 10.3390/cancers18121968

**Published:** 2026-06-17

**Authors:** Aaron Huang, Wayne C. Waltzer, Michael Hung, Frank S. Darras, Adam M. Kressel, Victor Romanov

**Affiliations:** Department of Urology, Renaissance School of Medicine, State University of New York at Stony Brook, Stony Brook, NY 11794, USA; aaron.huang@stonybrookmedicine.edu (A.H.); wayne.waltzer@stonybrookmedicine.edu (W.C.W.); michael.hung1@stonybrookmedicine.edu (M.H.); frank.darras@stonybrookmedicine.edu (F.S.D.); adam.kressel@stonybrookmedicine.edu (A.M.K.)

**Keywords:** bladder cancer, CIAO1, MALAT1, circular RNA, liquid biopsy

## Abstract

**Background and Objectives:** Bladder cancer (BCa) is characterized by high rates of recurrence and progression, underscoring the need for reliable non-invasive biomarkers. Circular RNAs (circRNAs) are covalently closed non-coding RNAs generated by back-splicing and are stable in biological fluids, including urine. Increasing evidence implicates circRNAs in BCa pathogenesis. However, identification of clinically relevant circRNAs remains challenging. This study aimed to streamline circRNA selection and identification of functional urinary circRNAs for potential use as biomarkers for BCa monitoring. **Methods:** Using a database-screening approach, we identified circRNAs with high predicted affinity (TDMD score > 1.1) to miR-101-3p (a tumor-suppressive microRNA in BCa). In addition, candidate circRNAs were prioritized based on the following: (i) derivation from genes involved in BCa tumorigenesis; and (ii) origination from exonic or long non-coding RNA sequences. The potential contribution of Argonaute-2 (Ago2) binding sites to circRNA activity or potential usage as biomarker was also evaluated. Expression levels were assessed in urine samples and BCa cell lines, and functional relevance was examined using molecular and cellular assays. **Results:** circCIAO1(5) and circMALAT1 fulfilled prioritization criteria and exhibited distinct Ago2-binding site profiles. Both circRNAs were upregulated in urine from BCa patients and in aggressive BCa cell lines and showed differential expression between remission and recurrent disease. CircCIAO1(5) demonstrated higher-affinity binding to miR-101-3p, while both circRNAs interacted with miR-101-3p and Ago2. Functional assays revealed enhanced proliferation, motility, and invasion upon circRNA overexpression, consistent with miR-101-3p sequestration and reduction in depression of its target oncogene—EZH2. **Conclusions:** circCIAO1(5) and circMALAT1 are promising candidates as urinary biomarkers for noninvasive BCa monitoring, illustrating the value of bioinformatics-guided determination of circRNA as potential biomarkers and significance of circRNA-mediated regulatory mechanisms in BCa biology.

## Simple Summary

Bladder cancer is a significant malignancy characterized by high rates of recurrence and progression, highlighting the need for reliable non-invasive biomarkers. Urine represents an optimal source of biomarkers for bladder cancer surveillance. Circular RNAs are a class of non-coding RNAs with a stable, covalently closed structure generated by back-splicing and are widely distributed in biological fluids. However, the identification and validation of clinically relevant circular RNAs remain labor- and time-intensive. This study aims to simplify circular RNA selection and identify functionally relevant candidates. Circular RNAs can regulate gene expression by acting as microRNA sponges. Using database-based screening, we identified two circular RNAs with high binding affinity for miR-101-3p, a known tumor suppressor in bladder cancer. Sequestration of miR-101-3p by these circRNAs enhanced cancer-related activity by limiting its interaction with key oncogenes. Importantly, both circRNAs exhibited distinct expression patterns in urine from bladder cancer patients compared with controls, supporting their potential utility as non-invasive biomarkers for disease monitoring.

## 1. Introduction

Bladder cancer (BCa) is a common and aggressive malignancy of the urinary tract, and current diagnostic tools such as cystoscopy and urine cytology remain either invasive or insufficiently sensitive, particularly for low-grade tumors. Therefore, developing reliable non-invasive biomarkers is a major clinical need. Several types of such biomarkers have been investigated including genomic, DNA-methylation, RNA and protein-based. Among RNA-originated biomarkers, microRNA molecules such as miR-6124 and miR-4511 have shown some promise [1,2]. Circulating nucleic acids are good candidates because of the relative simplicity and reliability of analysis [3,4,5]. In recent years, circular RNAs (circRNAs), a class of covalently closed non-coding RNAs generated through back-splicing of precursor mRNAs, have emerged as promising biomodulators in cancer research. Their high stability, tissue-specific expression, and presence in body fluids such as urine make them attractive candidates for biomarker development [6,7,8,9,10,11,12]. Functionally, many circRNAs act as microRNA (miRNA) sponges, modulating the activity of miRNAs and thereby regulating downstream gene expression [13,14,15,16,17]. Dysregulated circRNAs have been implicated in various aspects of cancer biology, including proliferation, invasion, metastasis, and therapy resistance [6,8,9]. Several circRNAs show potential as biomarkers for BCa; however, to our knowledge, there are no clinical studies evaluating circRNA as a noninvasive biomarker for BCa monitoring. Among numerous miRNAs involved in tumorigenesis, miR-101-3p has been recognized as a tumor suppressor in several cancers, including BCa. It regulates the expression of oncogenes, including EZH2, thereby influencing cellular proliferation, motility, and epithelial–mesenchymal transition [18,19,20]. Downregulation of miR-101-3p has been associated with more aggressive tumor behavior and a poorer prognosis in BCa. CircRNAs could bind and sequester miR-101-3p, therefore playing a significant role in BCa progression [20,21,22]. Circ-BCRC4 is differentially expressed in normal and BCa tissue. Its overexpression in BCa cells increased miR-101-3p level, which suppressed EZH2 expression in BC cells. However, the mechanism of which circ-BCRC4 influences miR-101-3p is unlikely to involve direct miR-101-3p sponging [4].

We employed a bioinformatic strategy to identify circRNAs originating from BCa-related parental genes and with strong binding affinity for miR-101-3p that potentially can act as a sponge. Selected candidates differ in their genomic origins: circCIAO1(5) is generated from exon 5 of the CIAO1 protein-coding gene, whereas circMALAT1 originates from the *MALAT1* long non-coding RNA (LncRNA), whose biological activity is mediated through RNA-based interactions [23,24,25]. CircRNAs derived from exons and lnc-RNAs are more likely than intron-derived circ RNAs to act as miRNA sponges.

The presence of Ago binding sites, short RNA sequences on circRNAs that are complementary to the “seed region” of specific miRNAs, suggests that Ago2 may play a role in circRNA action. However, circRNA–Ago interaction is primarily mediated via the miRNA, not direct Ago–RNA binding. Additionally, the number of Ago sites itself does not increase direct interaction with microRNA but can increase the functionality of circRNA–microRNA indirectly. Ago2 acts as a bridge and can recruit other Ago proteins that may play a role in circRNA action [26,27,28]. Therefore, the two selected circRNA candidates possess distinct numbers of Ago binding sites to compare indirectly their role in circRNA activity.

Both candidates were examined for their differential expression in urine samples of BCa patients and healthy individuals, as well as in BCa cell lines with varying tumorigenic potential. Additionally, we assessed their ability to bind miR-101-3p in vitro, to regulate oncogenic pathways, and to influence malignant cellular phenotypes. The present study aimed to assess the potential of circCIAO1(5) and circMALAT1 as non-invasive urinary biomarkers for BCa and to elucidate their mechanistic roles in BCa progression through miR-101-3p sponging. Furthermore, we examined how the binding affinity and the number of Ago2 binding sites influence the functional properties of these circRNAs. A deeper understanding of the circRNA–miRNA interplay may not only facilitate the development of reliable diagnostic tools but also provide novel insights into the molecular mechanisms driving BCa pathogenesis.

## 2. Materials and Methods

### 2.1. Selection of Potential Candidates from Databases

Potential circRNA targets of miR-101-3p were identified using the starBase database (miRNA–circRNA interaction module) [29]. CircRNAs with a Target-Directed miRNA Degradation (TDMD) score greater than 1.1 were considered as high-affinity binders. Among these, circRNAs whose parental genes exhibited differential expression between BCa tissue and normal urothelium (based on the *starBase* Pan-Cancer dataset, The Cancer Genome Atlas (TCGA), and published data) and originated from exons or lnc-RNA were selected as candidates.

To further investigate the potential influence of Ago2 binding sites on circRNA function, two circRNAs meeting these criteria were chosen for detailed analysis: circCIAO1(5) (containing 3 Ago2 binding sites) and circMALAT1 (containing 88 Ago2 binding sites).

### 2.2. Cell Culture

Human BCa cells (T24 and 5637) and human uroepithelial immortalized UROtsa cells were obtained from ATCC (Manassas, VA, USA). T24 and 5637 cells were cultivated in RPMI 1640 medium (Thermo Fisher Scientific, Waltham, MA, USA) containing 10% fetal bovine serum, and UROtsa cells were grown in DMEM (Gibco, Grand Island, NY, USA) containing 10% FBS [30]. All cells were cultured at 37 °C in a 5% CO_2_ incubator.

### 2.3. Patient Specimens

Urine samples and 8 matched tumors were collected from 145 patients diagnosed with BCa from March 2022 to September 2025. Participants were selected during the urological clinic visits with a primary diagnosis of BCa. In addition, urine specimens were obtained from 25 healthy donors from March 2022 to August 2025. Ninety-three of these patients were male. The age range of patients and healthy volunteers was 28–95 years. In 46% of cases, the tumor was stage Ta, in 30% T1, in 7% T2, and in some cases stage was not determined. Pathologic staging was determined by trained genitourinary pathologists.

Informed consent was obtained from all participants. The study was approved by the Institutional Review Board of Stony Brook School of Medicine.

### 2.4. RNA Isolation, cDNA Synthesis, and RT-qPCR

RNA was extracted from tumors and cells with TRIzol (Thermo Fisher Scientific, Carlsbad, CA, USA) as described by the manual. RNA from urine was extracted with a Urine Cell-free circulating RNA kit (Norgen Biotech, Thorold, ON, Canada) or by using the protocol for nucleic acid preparation (CleanNA, Waddinxveen, The Netherlands) adjusted in our laboratory for the isolation of free nucleic acids from urine. Briefly: (i) urine was centrifuged at 3000× *g* for 10 min and supernatant was used for sample preparation; (ii) 20 µL of magnetic beads (CleanNGS) and 3 mL of Buffer K (Norgen Biotech, Canada) were added to 10 mL of urine and incubated on a rocking platform for 30 min at RT; (iii) beads with bound nucleic acid were washed 5 times with 1 mL of 75% EtOH; (iv) nucleic acids were eluted with 30 ul of water and (v) RNA was then purified using miRNeasy Mini Kit (Qiagen, Germantown, MD, USA) and eluted in 15 μL of water. After that, RNA was reverse transcribed into cDNA with the Evo cDNA kit (BioVision, Milpitas, CA, USA) using random primers, dT primers for control, or stem-loop primer: GTCGTATCCAGTGCAGGGTCCGAGGTATTCGCACTGGATACGACTTCAGT for miR-103-3p. All primers were synthesized by (IDT Coralville, IA, USA). One divergent primer for circRNA detection was designed by spanning the back-splicing junction (BSJ). This is especially critical for detection circRNA derived from a single exon. Reverse divergent primer was designed as described before [31]. Sequences of primers for RT-qPCR are provided (Table 1).

The circular nature of circCIAO1(5) and circMALAT1 was confirmed by Sanger sequencing encompassing the splicing junction site. RT-qPCR was performed with SYBR Green reagent (Abcam, Waltham, MA, USA) on the ABI 4300 system (Applied Biosystems, Wantham, MA, USA). U6 and β-Actin were used as internal references.

### 2.5. RNAS Treatment with RNase R

Isolated total RNA was treated for 30 min at 37 °C with or without 3 U/μL RNase R (Epicentre Biotechnologies, Madison, WI, USA). RNase R was inactivated at 65 °C for 20 min. Reaction buffer contained Li^+^ instead of K^+^ to improve the efficiency of linear RNA degradation. As was shown, this modification increased the ability of RNase R to proceed through these sequences and completely degrade the linear RNAs [32]. RNA was then purified using the RNeasy Mini Kit (Qiagen, Germantown, MA, USA) and eluted in 20 μL of water.

### 2.6. Molecular Cloning and Cell Transfection

CircCIAO1(5) siRNA was synthesized by IDT based on the sequence of mature circCIAO1(5)-CD.Ri.497024.13.1.

SEQ1 and SEQ2 CD.Ri.493950.13.1. Synthesized siRNAs encompassed junction area of circRNA. Scrambled DsiRNA (IDT, Coralville, IA, USA) served as non-related siRNA (siRNA NR). MiR-103-3p mimics were ordered from Qiagene (USA)—gene globe ID MSY0000099. MiR-101-3p Inhibitor was synthesized by IDT (USA).

CircRNA mini (mc2) expression plasmid (Addgene 206218) was used for cloning circCIAO1(5) and circMALAT1 between a pair of BsmB1 restriction sites [33]. CircCIAO1(5) was amplified by PCR with the following:

Forward primer: 5′-GCGTCTCA TCAG CTCTTAGCTTCTGCCAGCTA-3′

Reverse primer: 5′-TCGTCTCA TTAC TGTTCATTGCCTGGTAGATA-3′

circMALAT1 with the following:

Forward primer: 5′-GCGTCTCA TCAG AAACTTTGTCTGCGAACACT-3′

Reverse primer: 5′-TCGTCTCA TTAC CTAAAAATACACCAGCAAAA-3.

Amplification from cDNA performed with a high-fidelity DNA polymerase NEB Q5 DNA polymerase. The PCR amplicon is then digested with BsmBI, recovered using a DNA purification kit, and ligated with BsmBI-digested mc2 using T4 DNA ligase.

Plasmid DNA was obtained from bacteria grown overnight (37 °C, 230 rpm, 12 h) with the MiniPrep Qiagene kit (Germantown, MA, USA).

A total of 1 × 10^4^ 5637 or T24 cells were seeded into 24-well plates. Cells were reverse transfected with siRNAs or microRNA mimics with Lipofectamine RNAiMAX reagent (Invitrogen, USA). Plasmids containing circCIAO1(5) and circMALAT1 and the corresponding control (original mc2 vector) were transfected with Viafect (Promega, Madison, WI, USA). RT-qPCR was carried out after 24 h to examine the transfection efficiency.

### 2.7. Wound-Healing (Cell Motility) Assay

A wound-healing assay was performed as previously described [34]. Cells were cultured to 85–95% confluency in 24-well plates. The wound was made with a 200 µL pipette tip in 2 directions. Scratched cell monolayers were washed three times with PBS to remove detached cells, and 0.5 mL serum-free medium was added per well. Photographs of the cell wounds were taken with a microscope camera at 0, 18, and 24 h. Assay duration was adjusted to and minimize proliferation effects.

Wound area was calculated with Image ImageJ software, version 1.54 (NIH, Bethesda, MA, USA) as a percentage relative to negative control. The wound region was manually marked with the freehand selection tool and calculated with the “Measure” function. Images were analyzed under identical conditions.

### 2.8. Transwell Invasion Assay

For this assay, 100 μL 2 × 10^4^ cells in serum-free culture medium were plated into the upper chamber of the insert in a 24-well plate, which was precoated with Matrigel (BD Biosciences, Franklin Lakes, NJ, USA). A total of 600 μL medium with 10% FBS was added to the lower chamber. After 18 or 24 h of incubation, the cells that moved to the lower surface of the membrane were fixed with 4% paraformaldehyde and stained with 1% crystal violet. After removing cells from the upper part of the membrane, invaded cells on the lower part were treated with 10% acetic acid and absorbance at 525 nm of the extract was measured with a plate reader [31,35].

### 2.9. Dual-Luciferase Reporter Assay

Fragments of circCIAO1(5) and circMALAT1 wt., capable of binding to miR-101-3p as predicted with CircInteractome software (NIH, Bethesda, MD, USA) [36], and the EZH2 sequence that can potentially bind miR-101-3p as determined by miRDB software version 6.0 [37], as well as their mutated versions were inserted into pmiRGLO plasmid (Addgene 78131) [26,35] with single-nucleotide oligo bridge technology and the NEbuilder HiFi system (NEB, Ipswich, MA, USA) using NheI and XbaI restriction sites. After analysis, plasmids were transfected into BCa cells with or without miR-101-3p mimic using Viafect (Promega, Madison, WI, USA). Twenty-four hours later, firefly luciferase and Renilla luciferase were detected by using a Dual-Lumi™ Luciferase Assay Kit (Promega, USA). Assays were repeated three times.

### 2.10. RNA Immunoprecipitation (AGO2) Assay

Because AGO2 protein can interact with circRNAs and miRNAs, the RNA–protein complex can be precipitated with AGO2 antibody by RNA immunoprecipitation (RIP). The RIP assay was conducted with a RIP kit (Millipore, Sigma, St. Louis, MO, USA) according to the manufacturer’s instructions. Precipitated RNAs were detected by RT-qPCR [26].

### 2.11. Statistical Analysis

Relative gene expression was calculated using the 2^−^^ΔΔCt^ method, with normalization to the internal reference gene and calibration to the control group. Statistical analyses were performed on ΔCt values. Differences in ΔCt values between normal and tumor tissues were assessed using an unpaired two-tailed Student’s *t*-test (or Mann–Whitney U test, as appropriate). Correlation Pearson analysis was applied for paired urine–tissue samples. Data are presented as individual values with mean ± SD (or median with interquartile range). Experimental data were analyzed by GraphPad Prism 5.0.1 software. At least 3 independent experiments and 3 technical replicates were included in cell functional analysis. *p* value (*) < 0.05 was considered statistically significant.

## 3. Results

### 3.1. Bioinformatic Selection of Potential circRNA Biomarker Candidates

**Figure 1:**
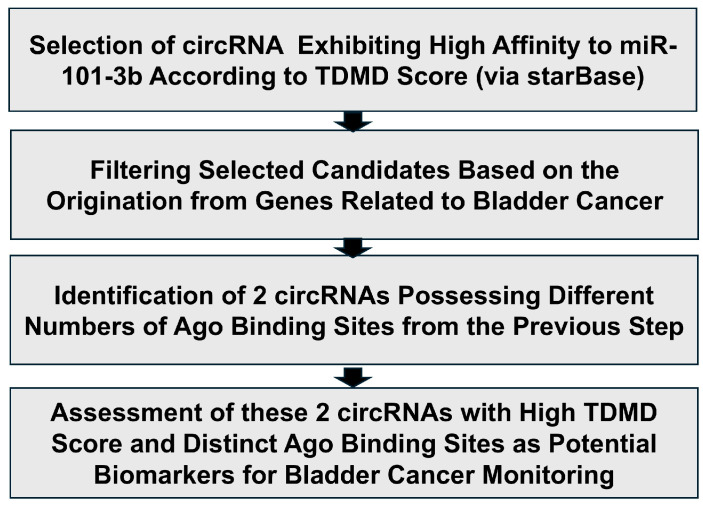
Workflow illustrates the bioinformatic pipeline for identification circRNA candidates with potential utility in BCa monitoring.

As shown in Figure 1 (upper box), the group of circRNA were selected based on the high binding affinity to miR-101-3p (TDMD score > 1.1) [29]. For this study, we considered circRNA derived from exons and lnc-RNAs. Inside this group, circRNA derived from genes related to BCa progression were determined and used for further analysis (Figure 1, second box).

This assessment was based on the differential expression of genes in tumor and normal urothelium (TCGA database reports amplification of CIAO1 in 3% tested cases, while MALAT1 in 2%). The starBase Pan Cancer option reported a difference of CIAO1 expression of 4.25 for cancer versus 3.7 (Log2) for normal urothelium. For MALAT1 expression, the difference was reported as insignificant in starBase. Nevertheless, amplification of this gene in BCa tissue compared to normal urothelium was reported in the literature [38,39]. CircCIAO1(5) originated from exon 5, and circMALAT1 from lnc-RNA.

Since we also aimed to examine the role of Ago binding to successful biomarker candidate selection, two candidates with different numbers of Ago binding sites from the group of circRNA that met the described requirements were chosen. Therefore, circCIAO1(5) and circMALAT1 qualified for the current study and were selected for validation as biomarkers for BCa monitoring and for the functional study (Figure 1 box 4).

### 3.2. Characterization of CircCIAO1(5) and circMALAT1 in BCa and Urothelial Carcinoma Cell Lines

CircRNA hsa_circ_0055631, designated as circCIAO1(5) by new classification [40], was formed through back-splicing of exon 5 of the CIAO1 gene on human chromosome 2, whereas hsa_circ_0002082 (circMALAT1) was formed through back-splicing of long non-coding RNA (lncRNA) of the MALAT1 gene on human chromosome 11. Since there are no clear guides about naming circRNA derived from non-coding RNAs [40], we selected the label circMALAT1 for hsa_circ_0002082 in this study.

**Figure 2:**
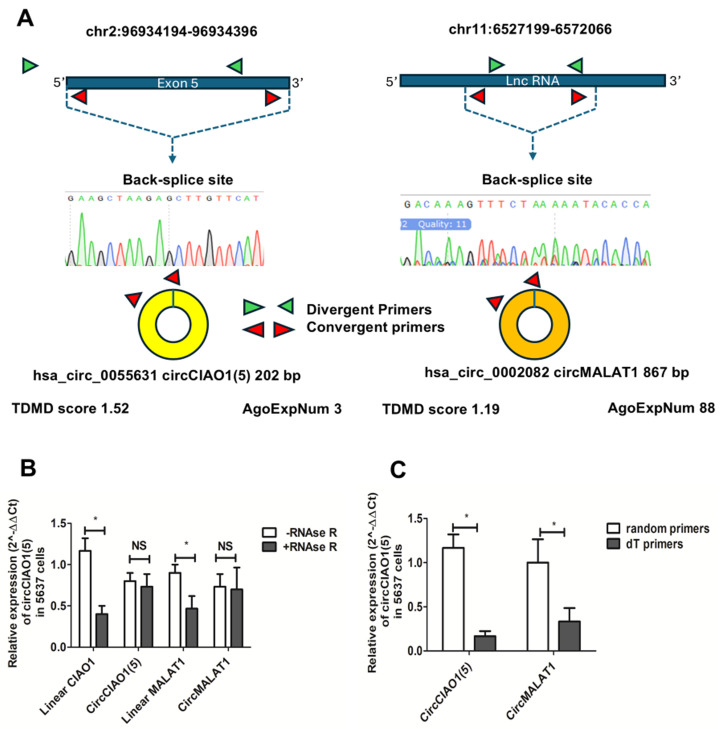
Identification and characterization of circCIAO1(5) and circMALAT1. (**A**) Schematic diagram of circCIAO1(5) and circMALT1 and Sanger sequencing of back-spliced site, (**B**) RNase R treatment, (**C**) reverse transcription with dT and random primers. * *p* < 0.05, NS-non significant.

Sanger sequencing validated the back-splicing site of circCIAO1(5) and circMALAT1 (Figure 2A).

RNase R treatment assays demonstrated the enhanced stability of circCIAO1(5) and circMALAT1 compared to the corresponding mRNAs (Figure 2B).

In addition, comparison of RT-qPCR results obtained using either an oligo(dT) primer or a random primer for reverse transcription revealed that circCIAO1(5) and circMALAT1 were expressed markedly higher when random primers were used (Figure 2C). This primer-dependent difference also supports the circular nature of analyzed RNAs.

### 3.3. CircCIAO1(5) and circMALAT1 Are Expressed at Higher Levels in Urine from Patients with Recurrent and Progressive Tumors

**Figure 3:**
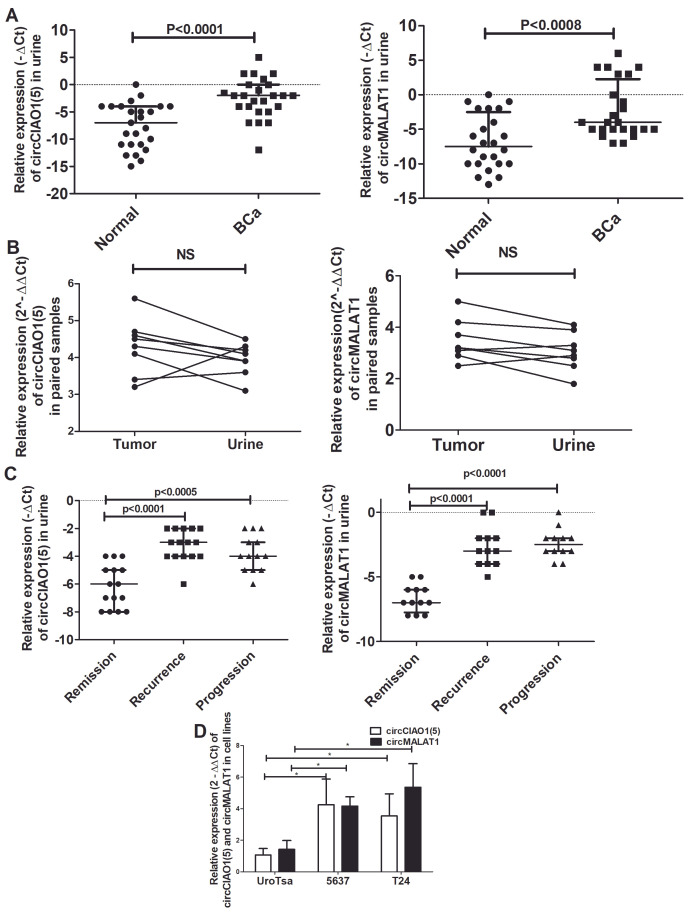
CircCIAO1(5) and circMALAT1 in BCa. (**A**) Relative CircCIAO1(5) and circMALAT1 expression in the urine of BCa patients and control group (25 samples in each group). (**B**) circCIAO1(5) and circMALAT1 expressions in pairs of urine–tissue samples and for circCIAO1(5) and 6 for circMALAT1, (**C**) circCIAO1(5) and circMALAT1 are expressed at higher levels in urine samples related to recurrent and progressive tumor. (**D**) circCIAO1(5) and circMALAT1 expression in BCa cell lines, NS—non-significant difference, data are shown as the means and SD of three independent experiments, * *p* < 0.05. Statistical analyses were performed on ΔCt values. For graphical representation expression was expressed as −ΔCt values or as fold change (2^−ΔΔCt^). NS-non significant.

Expression of circCIAO1(5) in BCa patient urine was significantly higher compared with urine from control: 4 cycles for circCIAO1(5) and 2.5 for circMALAT1 (in −ΔCt, N = 25). For several samples, RT-qPCR results were unreliable, likely due to low RNA quality or quantity (Figure 3A).

To determine whether the urinary circRNA originated from the bladder tumor, we quantified circCIAO1(5) and circMALAT1 in eight paired bladder tumor tissues and corresponding urine samples. CircCIAO1(5) was detected in seven pairs; in one case, the urinary circCIAO1(5) level was below the detection threshold, whereas circMALAT1 was detected in six pairs (Figure 3B). No significant difference was observed between tumor and urine originating samples (r = 0.3, *p* = 0.32 for circCIAO1(5) and r = 0.12, *p* = 0.7 for circMALAT1) by Pearson analysis. Wilcoxon matched-pairs signed rank testing also did not show significant differences.

To assess whether circCIAO1(5) and circMALAT1 expression correlated with clinical status, we analyzed their levels in urine from patients with distinct clinical courses. Each BCa patient was classified into one of three groups based on their clinical follow-ups: remission, recurrence, or progression. Remission was defined as the absence of detectable BCa during surveillance following surgery. Recurrence was defined as the reappearance of BCa of the same or lower grade and stage during surveillance. Progression was defined as the development of a tumor with higher grade or stage compared with the initial diagnosis. Twelve age-matched samples (55–70 years) were selected for each group.

CircCIAO1(5) levels were 3 cycles higher in urine from patients with recurrent tumors and 2 cycles higher in those with progressive disease compared with patients in remission. CircMALAT1 level was 5 cycles higher in the recurrence and 5.5 higher in progression groups (Figure 3C) (in −ΔCt, N = 12). We next examined circCIAO1(5) and circMALAT1 expression in urothelial cell lines. Both circRNAs showed elevated expression in the aggressive BCa cell lines 5637 and T24 compared with the non-transformed urothelial line UROtsa. A non-related circRNA was used as a negative control for the comparison (Figure 3D).

### 3.4. Knockdown of circCIAO1(5) and circMALAT1 Inhibits BCa Cell Proliferation, Motility, and Invasion While Their Overexpression Promoted These Functions

**Figure 4:**
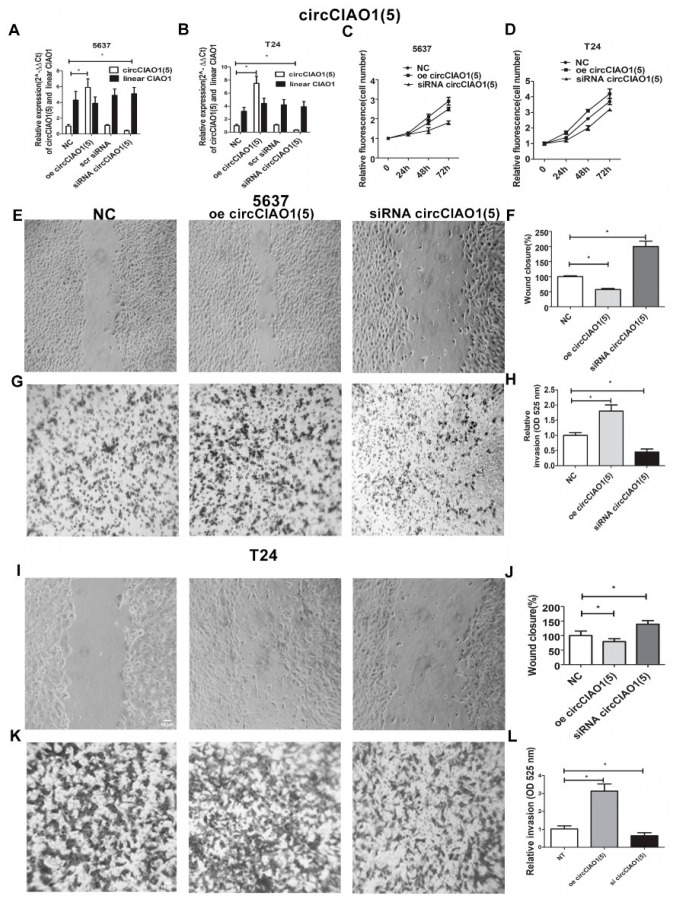
Effect of CircCIAO1(5) on proliferation, motility, and invasion of BCa cells. CircCIAO1(5) in 5637 (**A**) and T24 (**B**) cells following overexpression of circCIAO1(5) or siRNA-mediated silencing; linear CIAO1 RNA served as a control. (**C**,**D**) Proliferation of 5637 or T24 cells upon modulation of circCIAO1(5). (**E**,**F**) Representative images and quantification illustrating 5637 cell motility upon modulation of circCIAO1(5). (**G**,**H**) Representative images and quantification of 5637 cell invasion showing dependence on circCIAO1(5). (**I**,**J**) Representative images of cell motility demonstrating circCIAO1(5) dependent regulation in T24 cells. (**K**,**L**) Representative images and quantification of T24 cell invasion showing dependence on circCIAO1(5). qPCR data were analyzed using the ΔΔCt method. Statistical analyses were performed on ΔCt values. For graphical representation, expression was expressed as fold change (2^−ΔΔCt^) for interpretation. Data is presented as means ± SD from three independent experiments. * *p* < 0.05. Scale bar on the bottom of left image (50 μm) is applied to all images.Start point images in scratch assay for both cell lines are on Supplementary Figure S1.

**Figure 5:**
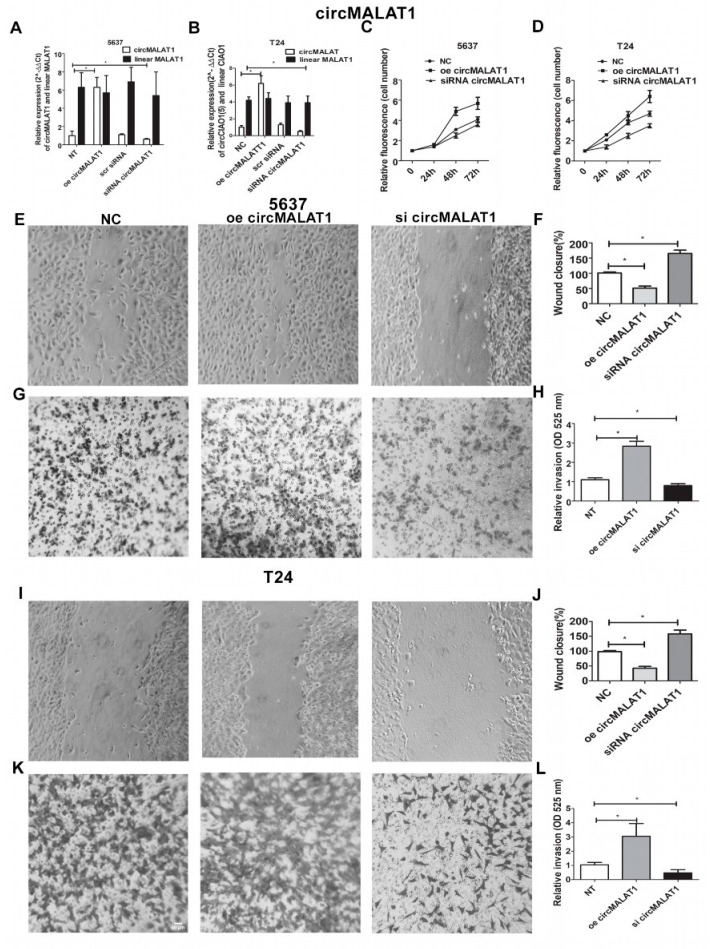
Effect of circMALAT1 on proliferation, motility and invasion of BCa cells. CircMALAT1 in 5637 (**A**) and T24 (**B**) cells following overexpression of circMALAT1 or siRNA-mediated silencing; linear RNA served as a control. (**C**,**D**) Proliferation of 5637 or T24 cells upon modulation of circMALAT1. (**E**,**F**) Representative images and quantification illustrating 5637 cell motility upon modulation of circMALAT1. (**G**,**H**) Representative images and quantification of 5637 cell invasion showing dependence on circMALAT1. (**I**,**J**) Representative images of cell motility demonstrating circCIAO1(5) dependent regulation in T24 cells. (**K**,**L**) Representative images and quantification of T24 cell invasion showing dependence on circMALAT1. RT-qPCR data were analyzed using the ΔΔCt method. Statistical analyses were performed on ΔΔCt values. For graphical representation, expression was expressed as fold change (2^−ΔΔCt^) for interpretation. Data is presented as means ± SD from three independent experiments. * *p* < 0.05. Scale bar on the bottom of left image (50 μm) is applied to all images.Start point images in scratch assay for both cell lines are on Supplementary Figure S1.

To investigate the biological roles of circCIAO1(5) and circMALAT1 in BCa, we designed two siRNAs targeting the back-splice junctions of circCIAO1(5) and circMALAT1 (see Section 2) and transfected them into 5637 or T24 cells. We also constructed overexpression plasmids by inserting the circRNA-producing exons between intronic sequences previously shown to enhance back-splicing. RT-qPCR confirmed that transfection of these plasmids increased circCIAO1(5) (Figure 4A,B) and circMALAT1 (Figure 5A,B) expression, whereas siRNAs efficiently reduced circCIAO1(5) (Figure 4A,B) and circMALAT1 (Figure 5A,B) expression by several fold in both cell lines. Importantly, the expression of the corresponding linear mRNAs remained largely unchanged following either siRNA-mediated knockdown or plasmid-driven overexpression (Figure 4A,B or Figure 5A,B).

We next assessed the impact of circCIAO1(5) and circMALAT1 expression on BCa cell behavior, including proliferation, motility, and matrix invasion. In 5637 and T24 cells, siRNA-mediated silencing of circCIAO1(5) (Figure 4C,D) and circMALAT1 (Figure 5C,D) significantly reduced cell proliferation, whereas overexpression of either circRNAs enhanced proliferation.

In addition, overexpression of circCIAO1 (Figure 4E,F,I,J) and circMALAT1 (Figure 5E,F,I,J) enhanced cellular motility, whereas knockdown of either circRNA exerted a more pronounced effect, leading to a significant suppression of this activity in both cell lines. Matrix invasion increased following circCIAO1(5) (Figure 4G,H,K,L) or circMALAT1 (Figure 5G,H,K,L) overexpression and was reduced upon transfection with the corresponding siRNAs into 5637 and T24 cell lines.

### 3.5. CircCIAO1(5) and circMALAT1 May Act as a Sponge for miR-101-3p in BCa Cells

**Figure 6:**
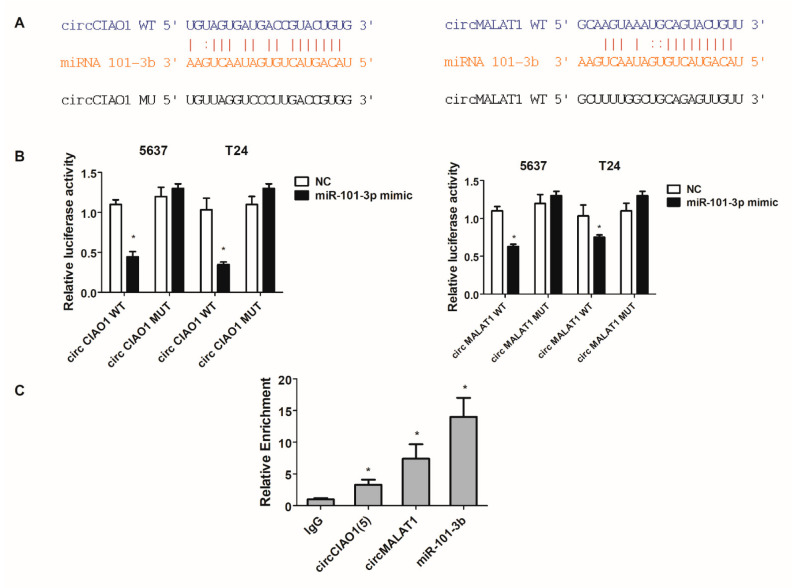
Interaction of circCIAO1(5) and circMALAT1 with miR-101-3p. (**A**) Predicted miR-101-3p binding motifs within circCIAO1(5) and circMALAT1. (**B**) Dual-luciferase reporter assays demonstrating the interaction between circCIAO1(5) (**left**) or circMALAT1 (**right**) and miR-101-3p. (**C**) AGO2 RNA immunoprecipitation (RIP) showing enrichment of circRNAs and miR-101-3p in AGO2 complexes. Data is presented as means ± SD from three independent experiments. * *p* < 0.05.

Bioinformatic analysis identified putative miR-101-3p binding sites within circCIAO1(5) and circMALAT1 (Figure 6A). To validate these interactions, luciferase reporter assays were performed using constructions containing the predicted binding regions or their corresponding mutant sequences. In 5637 and T24 cell lines, co-transfection with miR-101-3p mimics significantly decreased luciferase activity of the wild-type reporters, whereas no reduction was observed for the mutant constructions. Luciferase activity of the circCIAO1(5) reporter decreased by approximately 60%, compared with a ~30% reduction for the circMALAT1 reporter (Figure 6B), supporting differential binding affinity of the two selected circRNAs for miR-101-3p.

Further evidence supporting circRNA–miRNA interaction was obtained using AGO2 RNA immunoprecipitation (RIP) in 5637 cells. qRT-PCR analysis demonstrated a marked enrichment of circCIAO1(5) and circMALAT1 in AGO2 immunoprecipitated relative to IgG controls. Notably, circMALAT1 showed higher enrichment than circCIAO1(5), which is consistent with its greater number of predicted AGO2-binding sites (88 versus 3). miR-101-3p was also robustly enriched in AGO2 pulldown, reaching levels exceeding those of circMALAT1, likely to reflect the generally higher abundance of miRNAs relative to circRNAs in cells (Figure 6C).

### 3.6. miR-101-3p Expression Is Correlated with BCa Progression and Suppresses Proliferation, Migration, and Invasion of BCa Cells

**Figure 7:**
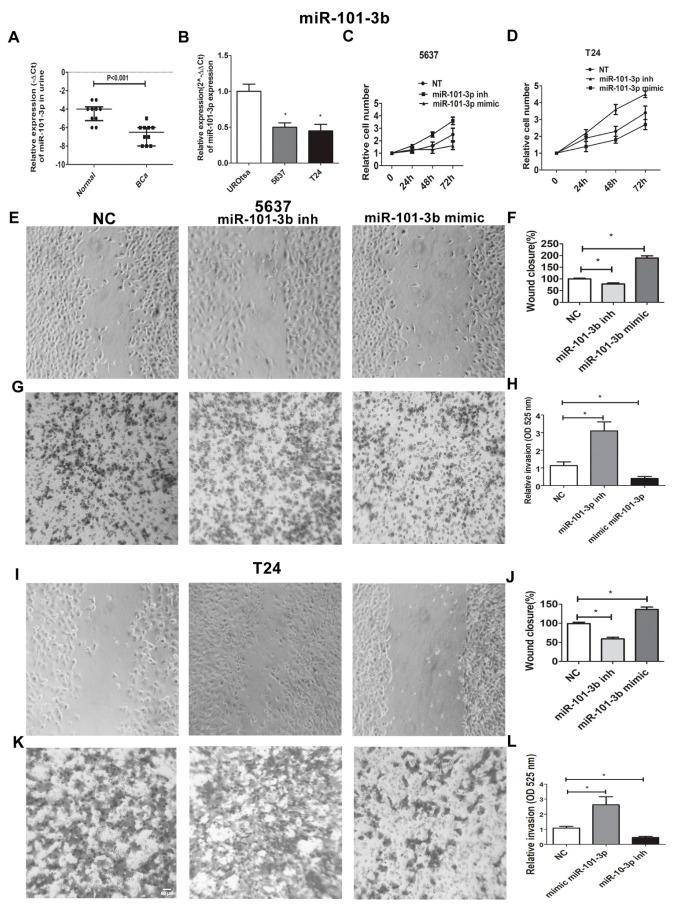
miR-101-3p expression is elevated in BCa and regulates proliferation, motility, and invasion of BCa cells. (**A**) miR-101-3p expression levels in urine samples from BCa patients and controls. (**B**) miR-101-3p expression in urothelial and BCa cell lines. (**C**) Effects of miR-101-3p modulation on proliferation of 5637 cells. (**D**) Effects of miR-101-3p modulation on proliferation of T24 cells. (**E**,**F**) Regulation of 5637 cell motility by miR-101-3p. (**I**,**J**) Regulation of T24 cell motility by miR-101-3p. (**G**,**H**) Regulation of 5637 cell invasion by miR-101-3p. (**K**,**L**) Regulation of T24 cell invasion by miR-101-3p. Data is presented as means ± SD from two independent experiments. * *p* < 0.05. Scale bar on the bottom of left image (50 μm) is applied to all images.Start point images in scratch assay for both cell lines are on Supplementary Figure S1.

Using RT-qPCR, we found suppressed expression of miR-101-3p in the urine of BCa patients and in more aggressive BCa cell lines (Figure 7A,B).

Proliferation of 5637 (Figure 7C) or T24 cells (Figure 7D) was modulated by miR-101-3p expression. Mobility of 5637 (Figure 7E,F) and T24 (Figure 7I,J) increased when miR-101-3p inhibitor was expressed while transfection with miR-101-3p mimic impaired it. For 5637 (Figure 7G,H) or T24 cells (Figure 7K,L), a trend of increasing invasion after transfection with miR-101-3p inhibitor and decreasing with miR-101-3p mimic was observed.

### 3.7. miR-101-3p Suppressed BCa Cell Proliferation, Migration, and Invasion by Targeting EZH2

**Figure 8:**
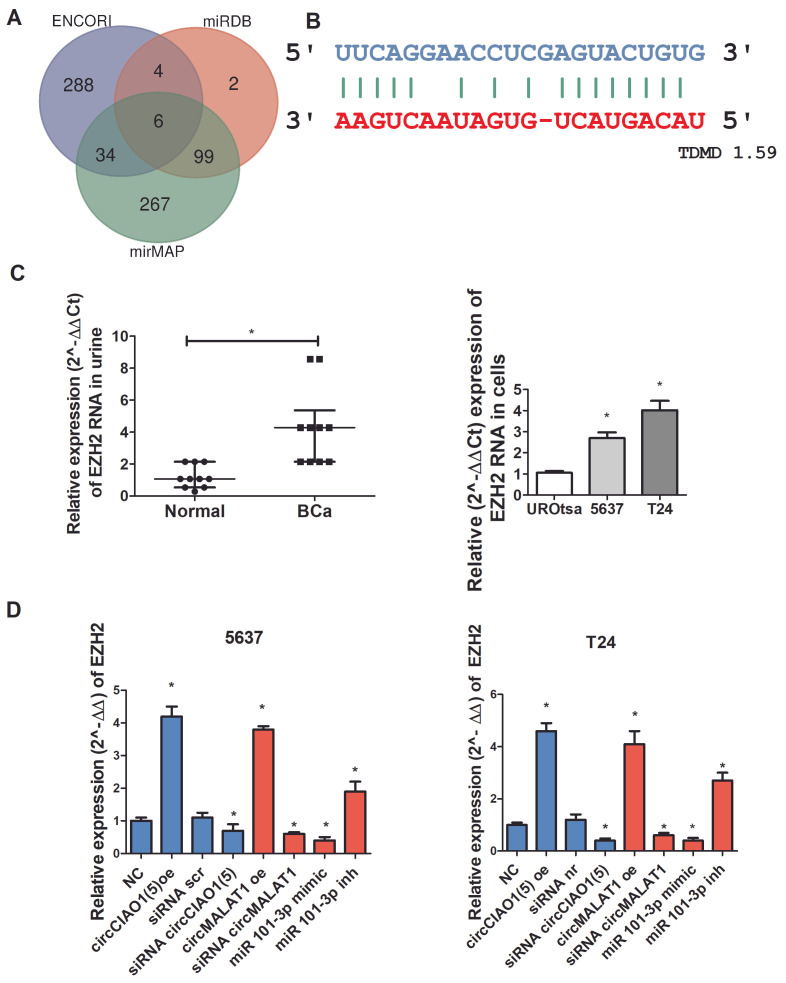
EZH2 is regulated by circCIAO1(5) and circMALAT1 through miR-101-3p. (**A**) Venn diagram for bioinformatic search of miR-101-3p binders. (**B**) Predicted miR-101-3p binding motifs within the 3′UTR of EZH2, indicating its potential regulation by miR-101-3p. (**C**) Relative EZH2 mRNA expression in urine samples from BCa patients, controls and in urothelial and BCa cell lines. (**D**) Regulation of EZH2 mRNA expression by circCIAO1(5) (blue bars), circMALAT1 (red bars), and miR-101-3p in 5637 and T24 cells. For ((**C**) left) data presented as median with interquartile range, for ((**C**) right) and (**D**), data are presented as means ± SD from three independent experiments. * = *p* < 0.05, for (**D**) *—compared with NC.

MiR-101-3p was identified as a tumor suppressor gene in BCa [19]. Candidate target genes of miR-101-3p were predicted using TargetScan, mirMAP and ENCORI. Top binders were selected by combined search (Figure 8A). Six genes—EZH2, LCOR, GPAM, CADM1, ZC3H11A, and TGFBR3—were present in all three databases. Further filtration of the list was achieved in analyzing expression alteration and role in survival in BCa (TGGA). Only ZC3H11 showed low amplification in BCa and was excluded. Survival analysis showed that only EZH2 and TGFBR alteration impaired survival in BCa. However, EZH2 emerged as the most biologically and clinically relevant candidate in BCa, supported by strong experimental validation and its established oncogenic role, and was therefore considered as a candidate for miR-101-3p binding and regulation [18,41,42,43].

### 3.8. CircCIAO1(5) and circMALAT1 Regulate EZH2 Expression in BCa Cells

We found that EZH2 was overexpressed in tumor urine samples as compared with control (Figure 8C) (−ΔCt difference 2.5 N = 10). EZH2 RNA levels were also elevated in BCa cell lines with higher malignant potency (Figure 8C). CircCIAO1(5) and circMALAT1 overexpression in 5637 and T24 cells resulted in increase in EZH2 mRNA levels. Conversely, siRNA-mediated circCIAO1(5) and circMALAT1 silencing led to a reduction in EZH2 expression (Figure 8D). Consistent with the known interaction between miR-101-3p and *EZH2*, miR-101-3p overexpression significantly decreased EZH2 expression, whereas inhibition of miR-101-3p activity induced an increase in EZH2 mRNA levels (Figure 8D).

## 4. Discussion

A common strategy for identifying circRNA biomarker candidates involves comparing circRNA expression profiles between normal and pathological samples and selecting transcripts that are differentially expressed between these groups. Although circRNA microarray profiling is a powerful discovery tool, it remains relatively costly, labor-intensive, and time-consuming. Moreover, commercial arrays include only predefined circRNA catalogs, limiting the identification of novel or less-characterized candidates [44,45,46,47].

One mechanism through which circRNAs exert regulatory functions is the sequestration (“sponging”) of functional miRNAs [20,48]. Although this mechanism is well established for a small number of circRNAs—most notably CDR1as, which contain more than 60 miR-7 binding sites [49,50]—transcriptome-wide studies suggest that most circRNAs rely on weaker and often noncanonical forms of miRNA engagement.

In this study, we pursued a streamlined bioinformatics-based selection strategy by evaluating possible interactions of circRNA with miRNA of interest (miR-101-3p) [14,15].

In BCa, miR-101-3p is widely recognized as a critical tumor-suppressive microRNA (specifically in urothelial carcinoma). Its downregulation represents a hallmark of aggressive disease, and this dynamic impacts several critical signal transduction pathways [51]. Decreased serum levels of miR-101-3p have been proposed as a viable diagnostic biomarker, correlating negatively with advanced clinical characteristics [52]. Because the present study focuses on the discovery of novel circular RNA (circRNA) interactions with miR-101-3p, it was essential that the literature confirms that other non-coding transcripts actively sponge this microRNA, inducing cancer-related changes in BCa [20,53]. Therefore, despite the dense network of miRNAs involved in bladder carcinogenesis, miR-101-3p was selected as the optimal target candidate for this investigation.

Candidate circRNAs were identified directly from public databases based on three criteria: (i) predicted high binding affinity to miR-101-3p; (ii) documented involvement of their parental genes in BCa development and progression; and (iii) exon or lnc-RNA origin of selected circRNA. Exonic circRNAs, which arise from protein-coding exons, are predominantly localized in the cytoplasm, where they can sequestrate miRNAs. Similarly, circRNAs derived from lncRNAs—such as those produced from MALAT1 or H19—may in some cases also exert sponge activity, provided they accumulate in the cytoplasm and harbor functional miRNA-binding motifs. These circRNA classes contribute to post-transcriptional regulation through miRNA sequestration, with their functional impact determined by circRNA abundance, subcellular localization, and the number and accessibility of embedded miRNA response elements [52,53]. Since many circRNA present in fewer copy numbers than microRNA, the sponging phenomenon still needs detailed confirmation.

To prove interactions of circCIAO1(5) and circMALAT1 with miR-101-3b in this study, we showed direct binding in vitro as well as coexistence with AGO2 protein.

We further examined the relevance of AGO-binding site abundance as a determinant of circRNA functionality and potential biomarker utility. AGO proteins play a central role in miRNA-mediated gene silencing by stabilizing miRNA–target interactions and facilitating base pairing with target transcripts, including circRNAs. The number of AGO-binding sites contributes to the avidity and stability of the circRNA–miRNA complex and may determine the extent to which a circRNA can sequester a given miRNA. Importantly, Ago2 enrichment on a circRNA does not necessarily indicate strong sponging capacity. Rather, such interactions may represent transient binding events, seedless low-affinity contacts, or protein-mediated recruitment mechanisms [54]. Consequently, circRNAs with limited predicted seed sites, but detectable AGO2 occupancy, may still modulate miRNA activity, because molecular sponging of tumor-suppressive miRNAs represents one of the key mechanisms through which circRNAs may influence cancer. Thus, AGO-binding site composition is an important parameter for prioritizing circRNAs for functional characterization [16,28].

Using this approach, we identified and validated circCIAO1(5) and circMALAT1 with different binding affinity, number of Ago2 sites, and gene origin in BCa cell lines, tumor tissues, and, critically, in urine samples from patients. To confirm the circular nature of these transcripts, we verified their back-splice junctions and demonstrated resistance to RNase R digestion, supporting their bona fide circular structure.

As no previous data existed regarding the expression of these circRNAs in normal urothelium, we quantified their levels in clinical urine specimens and observed higher expression of both circCIAO1(5) and circMALAT1 in samples from patients with recurrent or progressive disease. In most cases, these circRNAs were detectable in urine samples (15 mL) by conventional RT-qPCR. Two circRNAs examined here differ substantially in several properties: (i) predicted binding strength to miR-101-3p (TDMD scores: 1.52 for circCIAO1 and 1.19 for circMALAT1); (ii) number of Ago2-binding sites (3 for circCIAO1 and 88 for circMALAT1); (iii) transcript size (202 bp vs. 887 bp, respectively); and (iv) the nature of their parental genes (a protein-coding exon for circCIAO1 vs. lncRNA for circMALAT1). Both circRNAs showed potential use as biomarkers for predicting tumor progression. However, at this stage of the study it is difficult to conclude which circRNA characteristics are more important for the performance of the selected candidate as a biomarker or miRNA sponge. Integrating all database-annotated features of the candidate circRNAs will markedly improve the robustness of our selection strategy. Nonetheless, because these features overlap in function and predictive value, and only two candidates were considered, it was difficult to disentangle the relative contribution of each individual feature in this study. A future study will use more restrictive candidate selection and larger sample sets that will investigate the role of these characteristics separately.

Manipulating circCIAO1(5) and circMALAT1 expression in BCa cells demonstrated that several tumor-associated phenotypes depend, at least in part, on their expression levels. Functional assays showed that both circRNAs contribute to the regulation of cell proliferation, motility, and invasion, consistent with their involvement in BCa progression. The modest impact of circRNA overexpression, assuming a miRNA-sponging mechanism, may be attributed to a limited number of miRNA molecules available for sequestration.

To evaluate the functional impact of circRNA manipulation, we utilized two distinct bladder cancer (BCa) cell lines differing in genetic origin and baseline malignancy. The T24 line represents a highly aggressive, invasive Grade 3 transitional cell carcinoma with established cisplatin resistance. In contrast, the 5637 line is derived from a less aggressive Grade 2 carcinoma characterized by distinct metabolic activity and lower cisplatin resistance compared to T24.

Gene and miRNA profiling previously confirmed that these lines differ significantly in key pathways, including mitochondrial metabolism and cisplatin sensitivity. However, miR-101-3p expression difference was not highlighted in this study [55]. Our experiments also did not show difference in miR-101-3p expression in these lines. Therefore, circRNA perturbation did not yield divergent phenotypic outcomes. The primary mechanism evaluated was the potential sponging of miR-101-3p by the target circRNAs. While the architecture of competing endogenous RNA (ceRNA) networks can be highly complicated—differing pools of alternative circRNA sponges could theoretically alter sponging efficiency despite identical mR-101-3p abundance—we did not detect this in our experimental model.

These circRNAs were selected for experimental validation based on their predicted high-affinity interactions with the tumor-suppressive miRNA miR-101-3p. In line with this, miR-101-3p overexpression in BCa cells reduced expression of the oncogenic target EZH2, highlighting a mechanistic axis in which circCIAO1(5) and circMALAT1 may modulate EZH2 levels through miR-101-3p sequestration. The most well-characterized role of miR-101-3p in oncology is its direct targeting of EZH2. Notably, higher MALAT1 expression was detected in BCa and regulation of cisplatin resistance was linked to MALAT1 RNA binding (sponging) to miR-101-3p [21].

**Figure 9:**
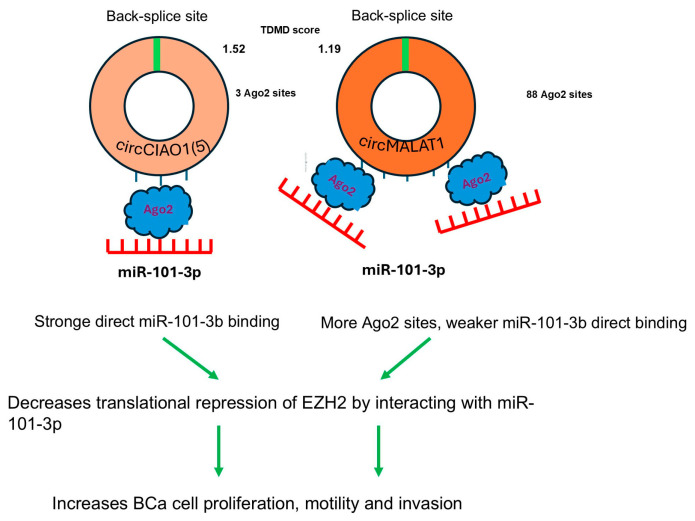
Schematic diagram for possible mechanism connecting circCIAO1(5) and circMALAT1 with miR-101-3p and EZH2 activity in BCa.

In this study, we also demonstrated that the expression of EZH2 is influenced by circCIAO1(5) and circMALAT1 levels (Figure 8). Together, circCIAO1(5), circMALAT1, miR-101-3p, and EZH2 form a regulatory network that may contribute to BCa cell proliferation, motility, and invasion (Figure 9).

Although our findings are preliminary, circCIAO1(5) and circMALAT1 show potential as non-invasive biomarkers for monitoring clinical status in BCa. Future studies incorporating longitudinal follow-up will be required to determine whether these circRNAs can reliably predict recurrence and progression.

## 5. Conclusions

This study revealed the tumor-promoting effects and potential mechanisms of action of circCIAO1(5) and circMALAT1. These candidates were chosen by a simple bioinformatic approach using publicly available tools. Elevated levels of these circRNAs in BCa specimens support their suitability as candidates for diagnostic or prognostic biomarkers. Collectively, these findings support the hypothesis that circCIAO1(5) and circMALAT1 may influence BCa cell behavior by modulating miR-101-3p activity. A reduction in the pool of free, functional miR-101-3p within cells may lead to suppression of EZH2, promoting a more malignant phenotype. Currently, no circRNA-based biomarker tests have received commercial approval, and the full extent of circRNA activity in gene regulation remains incompletely understood. Nevertheless, the inherent stability of circRNAs and their reliable detectability in biologically active fluids underscore their promise as a non-invasive biomarker candidate. Additionally, the use of RT-qPCR to detect these molecules in urine represents a highly feasible clinical diagnostic tool.

## Figures and Tables

**Table 1:** Primers for RT-qPCR.

Template		Primer 5′-3′	
circCIAO1 (5) BSJ	div	Forward	AATGAACAAGCTCTTAGCTTCTGC
hsa_circ_0055631		Reverse	TTCATTGCCTGGTAGATACTGAC
CIAO1	conv	Forward	AGGTCCTCCCTTCCCAGTTT
		Reverse	ATCCCCAGTTGCATCACAG
circMALAT1 a BSJ	div	Forward	GCTGGTGTATTTTTAGAAACTTTGTC
hsa_circ_0002082		Reverse	CCTTTTACTCTGATCATAATCTCCC
circMALAT1 b	div	Forward	CAGCTGAGTGATAAAGGCTGAG
hsa_circ_0002082		Reverse	AATTTGTCTTTCCTGCCTTAAAGT
MALAT1	conv	Forward	ACCTCTTAGACAGGTGGGAGA
		Reverse	TTAAAACCCCACAGGCACCC
EZH2	conv	Forward	TGTTTCTGTGTTCTTCCGCTT
		Reverse	CACTCCTTTCATACGCTTTTCTG
β-ACT	conv	Forward	CACCATTGGCAATGAGCGGTTC
		Reverse	AGGTCTTTGCGGATGTCCACGT
U6	conv	Forward	GCTTCGGCAGCACATATACTAAAAT
		Reverse	CGCTTCACGAATTTGCGTGTCAT
miR-101-3p	conv	Forward	TAGAGTACTGTGATAACTGAA
		Univ Reverse	CCAGTGCAGGGTCCGAGGTA

## Data Availability

The datasets generated and/or analyzed during this current study are not publicly available due to the need for further research but are available from the corresponding author upon reasonable request.

## Supplementary Materials

The following are available online at https://www.mdpi.com/article/10.3390/cancers18121968/s1, Figure S1: Images for scratch at starting points for all experimental conditions for both cell lines.
