# Supplementary figures and images for: Identification of circCIAO1(5) and circMALAT1 as Novel Potential Biomarkers for Bladder Cancer Monitoring Based on the Binding to miR-101-3p
Source: Cancers (Basel). 2026 Jun 17;18(12):1968. doi: 10.3390/cancers18121968 (PMC13297086; doi:10.3390/cancers18121968)

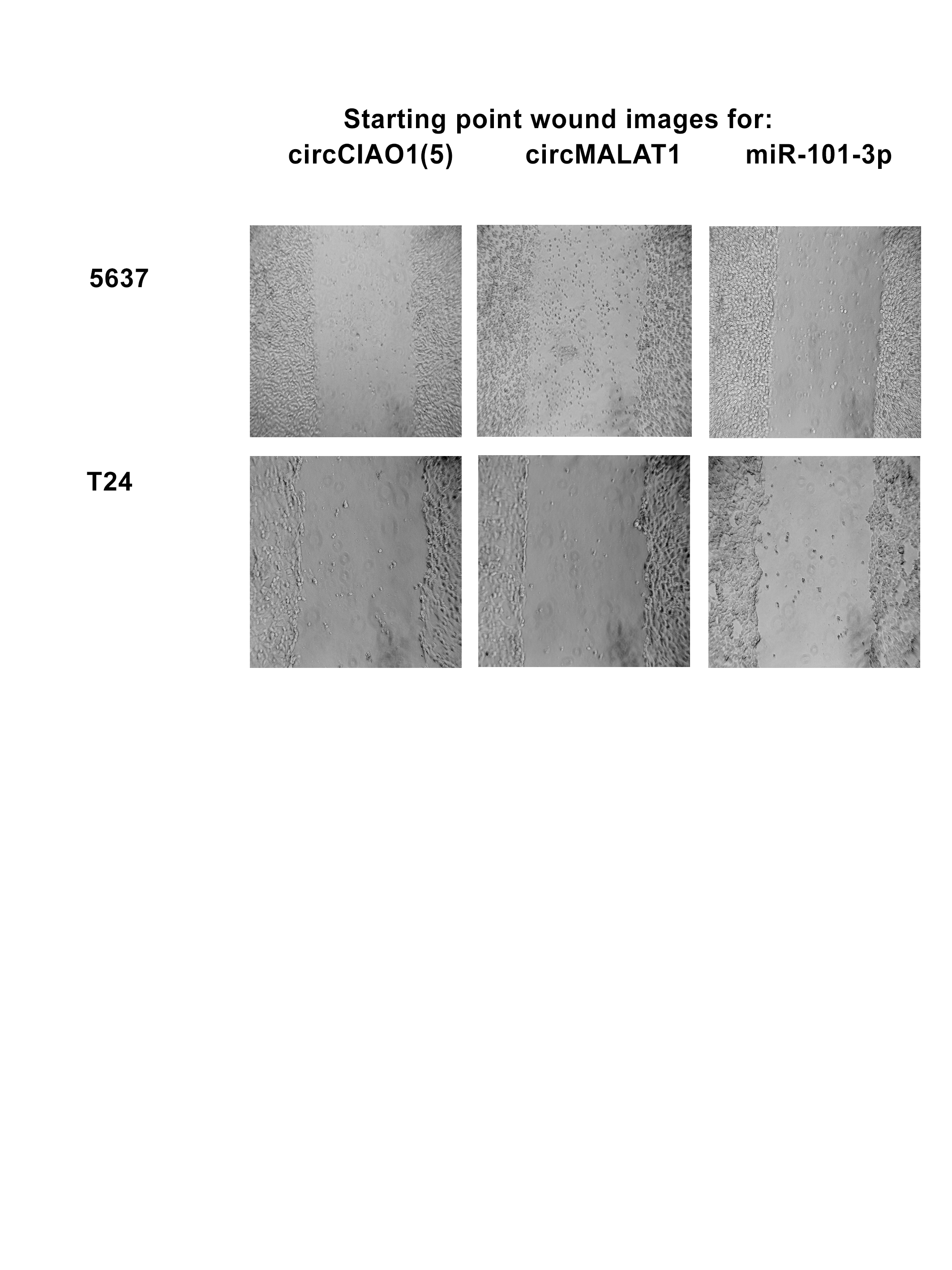

Supplement: Supplementary file 1 [file cancers-18-01968-s001.zip › cancers-4285107-Figure S1.tif]
